# The needs for diagnostic imaging in cases of group A streptococcal meningitis in children: a case report and review of the literature

**DOI:** 10.1099/acmi.0.000058

**Published:** 2019-08-19

**Authors:** Lise van Dijk, Tom F. W. Wolfs, Sylvia B. Debast, Veerle V. J. Langenhorst

**Affiliations:** ^1^​ Isala Hospital Zwolle, dokter van Heesweg 2, 8025 AB Zwolle, The Netherlands; ^2^​ University Medical Center Utrecht, Heidelberglaan 100, 3584 CX Utrecht, The Netherlands

**Keywords:** group A *streptococcus*, meningitis, cerebritis, cerebral complication, diagnostic imaging

## Abstract

Group A streptococcus (GAS) is a rare cause of bacterial meningitis in children and is associated with a high cerebral complication rate. In this case report, we present a 9-year-old girl with GAS meningitis complicated with cerebritis. Clear guidelines about choice of treatment and indications of follow-up by imaging tests are lacking, making GAS meningitis unpredictable and difficult to treat. Eventually, we found 25 paediatric cases of GAS meningitis presented in the literature and reviewed their treatment choices, outcomes and follow-up by imaging tests. Penicillin and ceftriaxone are most preferred for the treatment of GAS meningitis and adding rifampicin to the antibiotic treatment could be of potential benefit. When considering the duration of antibiotic treatment and follow-up by imaging tests, no clear recommendations were found. We found that GAS meningitis is associated with higher mortality and cerebral complication rates compared to other, more common, bacterial causes of meningitis in children. This should alert the clinician to consider imaging tests routinely, even if the patient improves clinically. We advise clinicians to routinely evaluate for possible cerebral complications through magnetic resonance imaging (MRI) scans. When cerebral complications are found, antibiotic treatment should be prolonged and adding rifampicin to the antibiotic regime may be considered.

## Introduction

Group A streptococcus (GAS), also known as *Streptococcus pyogenes *, is a causative agent of a wide variety of infections of the upper respiratory tract, skin and soft tissue, causing both invasive and non-invasive infections [1]. Acute pharyngitis, scarlet fever and erysipelas are well-known non-invasive GAS infections that tend to be more common and less severe [1]. Occasionally, GAS can cause invasive infections, which are less common, but more frequently associated with higher mortality and complication rates [1]. Meningitis as a manifestation of an invasive GAS infection is rare [2]. GAS accounts for less than 1 % of all bacterial cases of meningitis in children, with *Streptococcus pneumoniae *, *Neisseria meningitidis* and *Haemophilus influenzae* being the most common bacteria [1, 2].

## Case presentation

A previously healthy 9-year-old girl presented at the emergency department with suspected meningitis. She had been ill for several days with remitted fever and severe headache. There was no history of ear, nose or throat infection. At the time of presentation, she had a normal level of consciousness. She was febrile (38.7 °C, tympanic) with otherwise normal haemodynamic parameters. On physical examination, she revealed nuchal rigidity. Her skin showed no petechiae, purpura or rash and further neurological examination and examination of the ear, nose and throat (ENT), abdomen, heart and lungs revealed no abnormalities. The girl had received all vaccines according to schedule.

The laboratory results (see Table 1) showed an elevated total white blood cell count (TWBC) (43.2×10^9^ l^−1^) and a raised C-reactive protein (CRP) level (223 mg l^−1^). The haemoglobin (Hb) and thrombocyte counts were within normal ranges and except for low serum sodium (124 mmol l^−1^), the other serum electrolytes, renal and liver function tests showed no abnormalities. A lumbar puncture was performed and revealed turbid cerebrospinal fluid (CSF), with an elevated leukocyte count (1221×10^6^ l^−1^), low glucose (2.0 mmol l^−1^), a high total protein count (0.62 g l^−1^) and an elevated lactate level (9040 μmol l^−1^) (see Table 1). Further examination of the CSF by Gram stain revealed Gram-positive cocci with subsequent growth of β-haemolytic GAS that was sensitive to cephalosporin, penicillin, clindamycin and rifampicin. The blood culture was negative.

**Table 1.** Laboratory findings

Laboratory marker	Day 1	Day 17	Day 20	Reference
*Blood*				
CRP (mg l^−1^)	223	34	83	<10
TWBC (×10^9^ l^−1^)	43.2	13.1	10.8	4.5–11
*CSF*				
Leukocytes (×10^6^ l^−1^)	1221		89	<5.0
Glucose (mmol l^−1^)	2.0		3.2	2.1–4.2
Total protein count (g l^−1^)	0.62		0.78	0.18–0.58
Lactate level (μmol l^−1^)	9040		3440	1000–2000

CRP: C-reactive protein, CSF: cerebral spinal fluid; TWBC: total white blood cell count.

The patient was admitted to the paediatric ward when diagnosed with GAS meningitis. She was treated with ceftriaxone 100 mg/kg/day i.v. for 14 days and dexamethasone 0.6 mg/kg/day i.v. q.i.d. for 4 days. During admission, she temporarily developed hypothermia (35.5–36.5 °C) on days 1–3 and short-term (<24 h) diplopia and otalgia on day 2. Otherwise, she recovered quickly. Within 3 days, her headache had disappeared and her temperature had normalized. An audiogram was made and revealed no abnormalities. The patient recovered completely without neurological symptoms and she was discharged on day 7. Treatment with ceftriaxone i.v. was continued at home. The total duration of antibiotic treatment was 14 days.

Approximately 3 days after discontinuation of antibiotic treatment, the patient developed intermittent fever with moderate photophobia and hyperacusis. She was seen in the outpatient department on day 17. At that moment, she was febrile (39.6 °C, tympanic), but otherwise clinically in a fair condition. The haemodynamic parameters were within normal ranges and physical examination revealed no abnormalities, and in particular no nuchal rigidity. The laboratory results (see Table 1) still showed some slightly raised infection parameters, but they were much improved in comparison to those at the time of the previous admission. An intercurrent viral infection was considered and she was sent home. Imaging tests were not performed and watchful waiting was agreed.

**Fig. 1.**
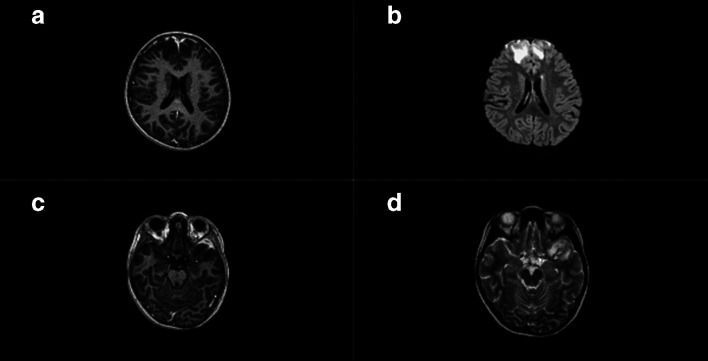
MRI imaging. (a) T1 gado: hypo intensity bilateral frontal with leptomeningeal enhancement. (b) FLAIR bilateral hyperintensity (vasogenic edema) in white matter and in subarachnoid space. (c) T1 gadolinium: enhancement of leptomeningeal with small empyema left middle cranial fossa. (d) T2 C involvement, left temporal lobe.

On day 20, however, she became delirious with fever, headache and vomiting. She was readmitted to the hospital. There was no nuchal rigidity and she was otherwise haemodynamically stable. To assess cerebral complications, a computed tomography (CT) scan and subsequently a magnetic resonance imaging (MRI) scan with contrast (see Fig. 1) were performed, and these showed signs of cerebritis with leptomeningeal enhancement of the frontal lobe bilaterally and the left temporal lobe without signs of raised intracranial pressure. A lumbar puncture was performed, which showed a slightly raised TWBC and protein count, and an elevated lactate level (see Table 1). The CRP level had increased to 83 mg l^−1^. The CSF and blood cultures remained negative. She was restarted on ceftriaxone 100/mg/kg/day. We added rifampicin 20 mg/kg/day orally. Her clinical condition improved quickly within 24 h and she was discharged after 9 days of treatment (i.e. day 29). Antibiotic treatment with ceftriaxone and rifampicin was continued at home for a total duration of 6 weeks. Follow-up imaging tests (i.e. an MRI scan) at the end of 5 weeks of antibiotic treatment (i.e. day 56) showed significant improvement. She recovered completely without neurological sequelae.

## Discussion

We describe a 9-year-old girl with GAS meningitis complicated with cerebritis. When considering this case, we searched for guidelines about the choice and duration of antibiotic treatment and indications for follow-up by imaging tests. Reviewing the literature, evidence-based guidelines and reviews about the treatment and follow-up of GAS meningitis in children are lacking. Eventually, we identified 25 published paediatric case reports about GAS meningitis from 1983 to 2018 with specifications about treatment, complications and follow-up [1–15]. An overview of these patients is given in Table 2. We added our case to the table. The age of the 25 paediatric patients varied between 2 weeks and 17 years. In 8 out of 25 patients, monotherapy with either penicillin (8/25) or ceftriaxone (3/25) was applied. In the other cases, combination therapy was preferred. It is unclear whether these antibiotics were initiated empirically or specified based on the CFS culture results. The duration of antibiotic treatment ranged from 10 to 84 days, with a median of 14 days. Imaging tests (i.e. CT and/or MRI scans) were performed for 9 patients, most of them in the context of suspected cerebral complications. Complications occurred in 56 % (14/25) of the patients, of which 64 % (9/14) contained cerebral involvement. Two patients died within 12 h after admission.

**Table 2.** Overview of 25 published cases of paediatric GAS meningitis

Author (reference)	Gender, age	Antibiotic treatment	Imaging	Complications
This case	Female, 9 years	Ceftriaxone i.v. 100 mg/kg/day, 14 days	CT and MRI on day 20, MRI on day 56	Cerebritis
van Zitteren *et al*. (2011) [1]	Female, 7 years	Penicillin i.v. 825.000 IU day^−1^, 14 days	None	None
Fanella & Embree (2008) [8]	Female, 14 years	Days 1–6: vancomycin i.v. 60 mg/kg/day, meropenem i.v. 120 mg/kg/day, ciprofloxacin* Days 6–42: penicillin i.v. 310.000 IU 6 h^−1^	CT on day of admission, on day 3, on day 9 and 1 month after discharge	Sinus venous thrombosis
Perera *et* *al*. (2005) [9]	Female, 6 weeks	Cefotaxime*, amoxicillin*, 14 days	None	None
Busetti *et* *al*. (2013) [2]	Male, 4 years	Ceftriaxone i.v. 1.5 g day^−1^, 10 days	None	None
Berner *et* *al*. (2000) [10]	Male, 7 years	Penicillin i.v. 300.000 IU day^−1^, 14 days	None	None
Paul and Jerwood (2012) [4]	Male, 4 years	Days 1–10: ceftriaxone i.v. 80 mg/kg/day, benzylpenicillin i.v. 50 mg/lg/day Days 11–22: cefotaxime i.v.*, clindamycin i.v.*	CT on day 12	Cerebral abscess
Bruun *et* *al*. (2010) [11]	Male, 8 years	Cefotaxime**, rifampicin**, clindamycin**, vancomycin**	CT^†^	Subdural effusion, hemiparesis and seizures
Pruva *et* *al*. (2004) [12]	Female, 2 years	Ampicillin**, ceftriaxone**, chloramphenicol**	None	None
Pruva *et* *al*. (2004) [12]	Female, 10 years	Penicillin**, ceftriaxone**	None	None
Shetty *et* *al*. (2001) [13]	Male, 3 years	Ceftriaxone* i.v. 100 mg/kg/12 h, vancomycin* i.v. 60 mg/kg/6 h	CT on day of admission	Cerebral edema, multiple infarctions and death (<24 h)
Moses *et* *al*. (1998) [14]	Male, 8 years	Penicillin**	CT on day of admission and 18 months after discharge	Seizures, cerebral edema, hygroma, encephalomalacia
Arnoni *et* *al*. (2007) [15]	Female, 5 years	Penicillin*, 10 days	CT follow-up^†^	Peripheral facial palsy
Arnoni *et* *al*. (2007) [15]	Male, 12 weeks	Ceftriaxone**	None	Septic shock and death
Sommer *et* *al*. (1999) [3]	Male, 15 years	Amoxicillin*, 21 days	None	None
Sommer *et* *al*. (1999) [3]	Female, 17 years	Ceftriaxone*, penicillin*, 21 days	None	None
Jagdis (1988) [18]	Female, 12 years	Penicillin 275.000 IU/kg/6 h, 13 days	CT on day of admission, on day 18 and 3 months after discharge	Cerebral abscess
Capua (2018) [19]	Male, 3 years	Ceftriaxone*, 12 weeks	CT on day of admission, MRI follow-up^†^	Cerebral abscess
Steppberger *et* *al*. (2001) [5]	Female, 11 years	Days 1–2: penicillin i.v.*, cefotaxime 2000 mg 6 h^−1^ Days 3–8: ceftriaxone 4000 mg 24 h^−1^ Days 3–12: fosfomycin 5000 mg 12 h^−1^	CT^†^	Internal third cranial nerve palsy and subdural hygroma’s
Murphy (1983) [6]	Male, 4 years	Day 1: ampicillin**, chloramphenicol** Days 2–10: penicillin G 250,000 U/kg/24 h	None	SIADH
Murphy (1983) [6]	Female, 8 years	Penicillin G 250,000 U/kg/24 h, 14 days	None	None
Murphy (1983) [6]	Male, 7 years	Days 1–3: ampicillin**, chloramphenicol** Days 4–18: penicillin G 250,000 U/kg/24 h	None	Seizures
Murphy (1983) [6]	Male, 10 years	Penicillin G 250,000 U/kg/24 h, 14 days	None	Psychomotor retardation, seizures, microcephaly and optic atrophy
Murphy (1983) [6]	Female, 12 years	Penicillin G 250,000 U/kg/24 h, 14 days	None	Bilateral hemianopsia
Murphy (1983) [6]	Female, 2 weeks	Penicillin**, gentamycin**	None	None
Walsh *et. al*. [7]	Female, 12 years	Ceftriaxone 1000 mg 12 h^−1^*, penicillin i.v. 1.5×10^6 ^4 h^−1^*	None	None

*dose or duration of treatment not available, **dose and duration of treatment not available, †specific day of imaging not available.

LP, lumbar punction.

SIADH, inappropriate secretion of anti diuretic hormone.

Reviewing the literature, remarkably high mortality rates and frequent short-term cerebral complications are reported in children with GAS meningitis. The major short-term cerebral complications described are cerebritis, cerebral edema and intracranial abscess. A Brazilian review of 71 published cases of paediatric GAS meningitis reported a mortality rate of 22 % and a total complication rate of 67 %, of which 8 % involved a brain abscess [16]. The authors did not otherwise differentiate cerebral from non-cerebral complications. Another review of 28 published paediatric cases found a mortality rate of 11 % over all cases and reported short-term cerebral complications such as brain abscesses, CSF leakage, subdural effusions and cavernous thromboses in 25 % of the 28 cases [13]. These findings are striking compared to those for other, more common, causes of bacterial meningitis in children. A meta-analysis including 4920 children with meningitis caused by either *S. pneumoniae *, *N. meningitidis* or *H. influenzae* reported mortality rates of 4–15 % and did not report on short-term cerebral complications. Cerebritis or brain abscess formation after meningitis caused by the more common micro-organisms is extremely rare.

With respect to the choice of antibiotic regime, penicillin and ceftriaxone are most frequently reported in the treatment of GAS meningitis. Penicillin penetrates well into the CSF and GAS continues to be exquisitely sensitive to penicillin [1]. However, despite this exquisite sensitivity of GAS to penicillin *in vitro*, *in vivo* this susceptibility is reduced by large bacterial loads. This is due to the downregulation of penicillin-binding proteins caused by the growth of GAS organisms [13]. Therefore, ceftriaxone could be a suitable alternative. Interestingly, recent studies have reported an increased efficacy of penicillin and ceftriaxone when rifampicin is added to the antibiotic regime. A pilot randomized controlled trial performed by Uppal *et al*. found significantly reduced markers of inflammation and neuronal damage in children with bacterial meningitis when pretreatment with a single dose of rifampicin was applied before the administration of ceftriaxone [17]. Hence in cases of invasive GAS meningitis where large bacterial loads can be expected, adding rifampicin to the antibiotic treatment with penicillin or ceftriaxone could be of potential benefit. With respect to the duration of antibiotic treatment, no recommendations were found in the literature.

### Conclusion

GAS is a rare cause of bacterial meningitis in children and is associated with high mortality and cerebral complication rates. Although it may have been overestimated because of publication bias, the high cerebral complication rate should alert clinicians to consider routine imaging tests, even when patients improve clinically. Our case clearly shows that clinical improvement alone does not exclude cerebral complications. We therefore advice clinicians to routinely evaluate for possible cerebral complications through MRI scans. When cerebral complications are found, antibiotic treatment should be prolonged and adding rifampicin to the antibiotic regime may be considered.
